# Rescue of a Plant Negative-Strand RNA Virus from Cloned cDNA: Insights into Enveloped Plant Virus Movement and Morphogenesis

**DOI:** 10.1371/journal.ppat.1005223

**Published:** 2015-10-20

**Authors:** Qiang Wang, Xiaonan Ma, ShaSha Qian, Xin Zhou, Kai Sun, Xiaolan Chen, Xueping Zhou, Andrew O. Jackson, Zhenghe Li

**Affiliations:** 1 State Key Laboratory of Rice Biology, Institute of Biotechnology, Zhejiang University, Hangzhou, China; 2 State Key Laboratory for Biology of Plant Diseases and Insect Pests, Institute of Plant Protection, Chinese Academy of Agricultural Sciences, Beijing, China; 3 Department of Plant and Microbial Biology, University of California, Berkeley, California, United States of America; Chinese Academy of Sciences, CHINA

## Abstract

Reverse genetics systems have been established for all major groups of plant DNA and positive-strand RNA viruses, and our understanding of their infection cycles and pathogenesis has benefitted enormously from use of these approaches. However, technical difficulties have heretofore hampered applications of reverse genetics to plant negative-strand RNA (NSR) viruses. Here, we report recovery of infectious virus from cloned cDNAs of a model plant NSR, Sonchus yellow net rhabdovirus (SYNV). The procedure involves *Agrobacterium*-mediated transcription of full-length SYNV antigenomic RNA and co-expression of the nucleoprotein (N), phosphoprotein (P), large polymerase core proteins and viral suppressors of RNA silencing in *Nicotiana benthamiana* plants. Optimization of core protein expression resulted in up to 26% recombinant SYNV (rSYNV) infections of agroinfiltrated plants. A reporter virus, rSYNV-GFP, engineered by inserting a green fluorescence protein (GFP) gene between the N and P genes was able to express GFP during systemic infections and after repeated plant-to-plant mechanical passages. Deletion analyses with rSYNV-GFP demonstrated that SYNV cell-to-cell movement requires the sc4 protein and suggested that uncoiled nucleocapsids are infectious movement entities. Deletion analyses also showed that the glycoprotein is not required for systemic infection, although the glycoprotein mutant was defective in virion morphogenesis. Taken together, we have developed a robust reverse genetics system for SYNV that provides key insights into morphogenesis and movement of an enveloped plant virus. Our study also provides a template for developing analogous systems for reverse genetic analysis of other plant NSR viruses.

## Author Summary

Reverse genetics is a powerful tool for fundamental studies of virus biology, pathology and biotechnology applications. Although plant negative-strand RNA (NSR) viruses consist of members in the *Rhabdoviridae*, *Bunyaviridae*, *Ophioviridae* families and several unassigned genera that collectively account for many economically important crop diseases, unfortunately, several technical difficulties have hindered application of genetic engineering to these groups of viruses. This study describes the first reverse genetics system developed for plant NSR viruses. We report an efficient procedure for production of infectious virus from cloned cDNAs of sonchus yellow net virus (SYNV) RNAs, a model plant rhabdovirus. We have also engineered a recombinant SYNV vector for stable expression of a fluorescent reporter gene. Using this system, we have generated targeted SYNV mutants whose analyses provide key insights into enveloped plant virus movement and morphogenesis processes. Moreover, our findings provide a template for reverse genetics studies with other plant rhabdoviruses, and a strategy to circumvent technical difficulties that have hampered these applications to plant NSR viruses.

## Introduction

Negative-strand RNA (NSR) viruses have major impacts on public health, agriculture and ecology, and they collectively are responsible for some of our most serious human, veterinary, wildlife and plant diseases [1]. Plant NSR viruses comprise members of the *Rhabdoviridae*, *Bunyaviridae*, *Ophioviridae* families, and of the unassigned *Emaravirus*, *Tenuivirus*, *Varicosavirus* and *Dichorhavirus* genera and account for many economically important crop diseases [1–3]. Most members of the plant NSR viruses are transmitted by specific arthropods (aphids, leafhoppers, thrips or mites) in which they also replicate, and many of these viruses share similarities in particle morphology, genome organization and fundemental replication strategies to their animal/human-infecting counterparts within the same families [3–7].

Generation of an infectious virus from a cDNA copy of the viral genome, an approach referred to as reverse genetics, is the most powerful genetic tool in modern virology. Unlike positive-strand RNA viruses, whose genomic RNAs (gRNAs) are infectious upon introduction into permissive host cells, neither the naked gRNAs nor the antigenomic RNAs (agRNAs) of NSR viruses are able to initiate infection process when present alone. Instead, replication initiation of these groups of viruses requires *de novo* viral mRNA synthesis from the viral nucleocapsid (NC) which consists of the viral gRNA and the NC core proteins [8,9]. Therefore, the minimal infectious units of NSR viruses are the viral NCs and generating infectious NCs for reverse genetic studies initially was a major challenge due to difficulties in *in vivo* reconstitution of functional NCs containing recombinant RNAs. Hence, nearly a decade passed after development of positive-strand virus reverse genetics systems before the first NSR reverse genetics applications were achieved with animal rhabdoviruses [10–12]. These successes involved an entirely different approach from that used to engineer positive-strand RNA viruses, and consisted of transforming cell lines expressing bacteriophage T7 polymerase with transcription plasmids encoding the core nucleocapsid proteins and exact copies of the agRNAs. Under these conditions, viable nucleocapsids were assembled *in vivo*, leading to replication of recombinant viruses in single cells, followed by invasion of surrounding cells to produce plaques that could be identified visually [10–12]. Notably, a key strategy leading to success was to express viral agRNAs rather than gRNAs, and it was thought that this circumvented hybridization of gRNAs and core protein mRNA transcripts to form double-stranded RNAs that could interfere with the template activities of the RNAs and trigger potent antiviral responses [13,14].

Since the initial rhabdovirus reverse genetics breakthroughs, related strategies have been developed for all families of animal NSR viruses, using either T7 polymerase or endogenous RNA polymerase I to direct intracellular transcription of exact copies of viral RNAs [15–20]. These accomplishments have permitted refined analyses of virus biology and pathology, construction of vectors capable of stable expression of foreign proteins, and attenuated recombinant virus vaccines [8,20–23]. Unfortunately, the inherent low efficiency of NSR virus rescue, coupled with several technical obstacles associated with plants, has hampered adaption of reverse genetics systems developed for animal/human NSR viruses to their plant counterparts during the past two decades. These problems include unavailability of plant or insect vector cell cultures suitable for virus replication and plaque formation, lack of T7 polymerase expression systems and poorly defined RNA polymerase I promoters in plants, as well as interference of the rigid plant cell wall with delivery of the multiple plasmids needed for NC reconstitution. Thus, the lack of reverse genetic systems for plant NSR viruses represents a critical technological gap that has severely hindered our understanding of plant NSR virus infection cycles and pathogenesis.

Plant rhabdoviruses are separated into the *Cytorhabdovirus* or *Nucleorhabdovirus* genera based on their cytoplasmic or nuclear sites of replication and morphogenesis, and all members have nonsegmented NSR genomes with a similar structural protein gene organization to those of animal rhabdoviruses [7]. Common elements of all rhabdovirus agRNA genomes consist of 5′ leader (le) and 3′ trailer (tr) sequences flanking five viral structural protein genes that are separated by gene junction sequences. Generally, the gene junction sequences are highly conserved within each virus and are moderately conserved amongst different rhabdoviruses. Three essential *cis*-elements are embedded in the gene junction sequences, i.e. the Gene-End elements that signal transcription termination and polyadenylation of upstream mRNAs, the Gene-Start elements for initiation of downstream mRNAs transcription and a non-transcribed intergenic region located between the Gene-End and Gene-Start elements. The five common rhabdovirus structural proteins consist of the nucleoprotein (N), phosphoprotein (P), matrix protein (M), glycoprotein (G), and the large RNA polymerase (L), organized in the order 5′-N-P-M-G-L-3′ on the agRNA. However, many rhabdovirus genera encode various additional accessory genes interspersed between the N and L genes [9]. The plant rhabdoviruses differ from their animal rhabdovirus counterparts by encoding one or more accessory movement proteins (MP), at least one of which is thought to be required for cell-to-cell movement [7]. *Sonchus yellow net virus* (SYNV), the most extensively studied *Nucleorhabdovirus*, encodes five structural proteins, plus sc4, a putative MP, in the order ‘N-P-sc4-M-G-L’. The sc4 protein is present in infected tissue, but does not form a major component of purified virus preparations [24]. During replication, the N, P and L core proteins assemble with viral gRNA or agRNA to form NCs that function in viral replication and transcription in the nuclei of SYNV-infected cells. As replication proceeds, the nuclei of SYNV-infected tissues become greatly enlarged and develop nuclear viroplasms [7,25]. During morphogenesis, the NCs presumably are coiled by the M protein to form bullet-shaped cores that bud through the inner nuclear envelopes to acquire host membrane lipids and viral glycoprotein spikes and accumulate as bullet-shape or bacilliform particles in perinuclear spaces [7,25]. Unfortunately, due to the lack of a reverse genetics system, none of the processes involved in replication, morphogenesis and cell-to-cell movement are well understood.

In this investigation, we describe for the first time the production of a recombinant plant NSR virus directly from cloned cDNAs. This system relies on co-infiltration of *Nicotiana benthamiana* leaves with *Agrobacterium tumefaciens* strains containing plasmids encoding the SYNV agRNA, the N, P and L core proteins, and viral suppressors of RNA silencing (VSRs). We have also engineered a reporter virus that can express green fluorescent protein (GFP) stably during several plant-to-plant passages. Deletion analyses with recombinant SYNV (rSYNV) have provided key insights into SYNV movement and morphogenesis. The establishment of SYNV reverse genetics provides a template for development of similar systems for other plant NSR viruses and will permit fundamental questions in plant NSR virus biology to be studied.

## Results

### Generation of infectious SYNV from cloned cDNAs

**Fig 1:**
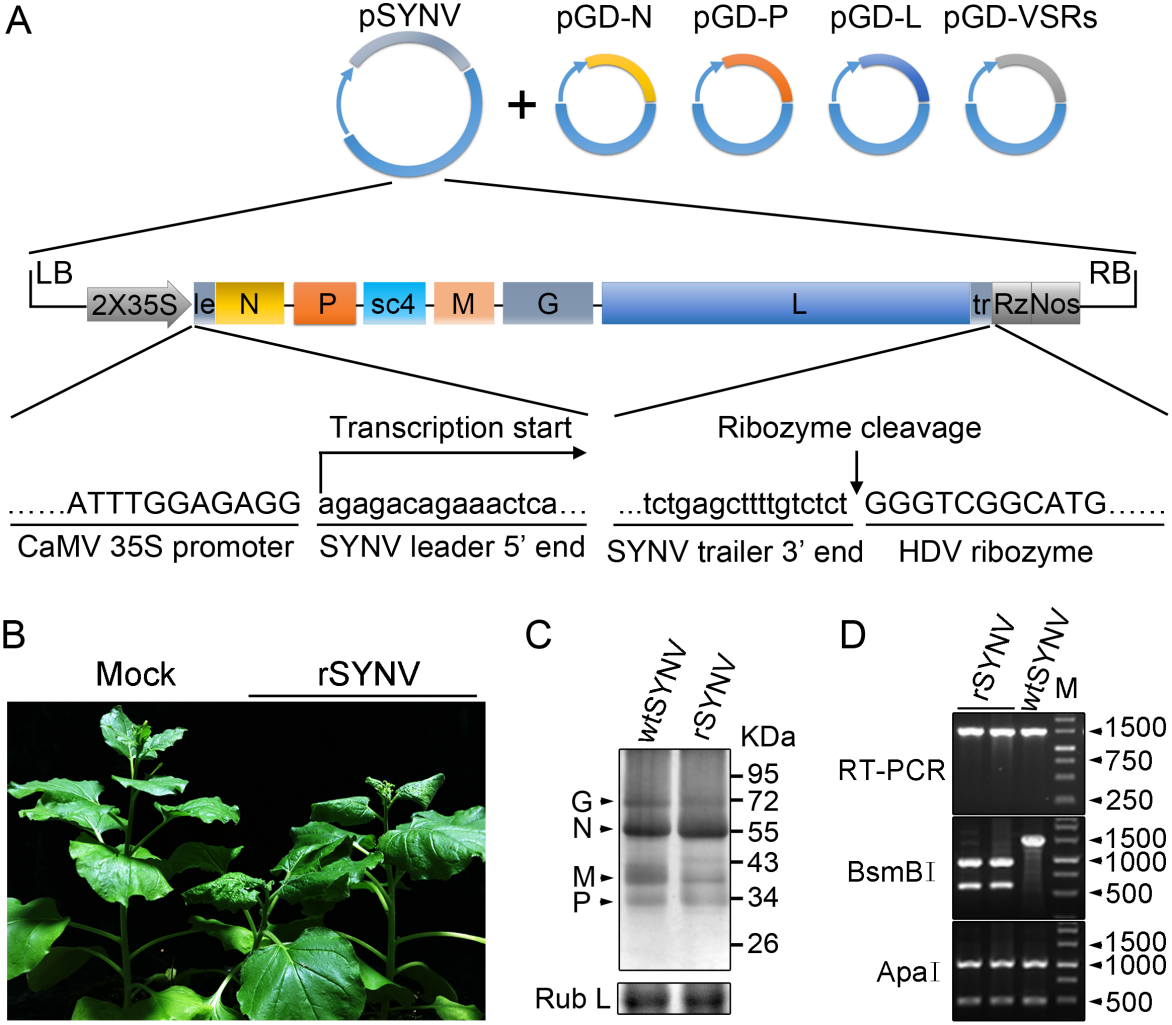
Recovery of recombinant SYNV in *N*. *benthamiana*. (A) Schematic representation of the SYNV infectious cDNA clone and supporting plasmids. The pSYNV plasmid is designed for transcription to yield the SYNV antigenome (ag) RNA and contains the full-length SYNV cDNA positioned between a truncated CaMV double 35S promoter (2X35S) and the HDV ribozyme sequence in the pCB301 plasmid. Note that the SYNV gene order is shown in the antigenome sense. The pGD-N, pGD-P and pGD-L supporting plasmids encode the viral N, P and L core NC proteins, respectively, and the pGD-VSRs encode the TBSV p19, BSMV γB, and TEV P1/HC-Pro suppressors of RNA silencing (VSRs). le: leader; tr: trailer; Rz: ribozyme; LB: left border sequence; RB: right border sequence; Nos: nopaline synthase terminator. (B) Symptoms of *N*. *benthamiana* plants systemically infected with rSYNV. *Agrobacterium* cultures containing the pSYNV, pGD-N, pGD-P, pGD-L and the three VSRs plasmids were mixed and infiltrated into *N*. *benthamiana* leaves. Infected plants showing stunting and typical vein clearing symptoms were photographed at 35 days post infiltration (dpi) along with a mock control (uninfected healthy plant). (C) Detection of viral structural proteins in wtSYNV- and rSYNV-infected *N*. *benthamiana* plants. Total protein samples were analyzed by Western blotting with anti-SYNV virion antibody. The Coomassie blue stained Rubisco large subunit (Rub L) was used as a loading control. The positions of the SYNV G, N, M and P proteins are indicated along the left side of the gel and the numbers along the right side are protein size markers in KDa. (D) Restriction enzyme site identification of wtSYNV and rSYNV cDNAs. Total RNAs were extracted from wtSYNV- and rSYNV-infected plants and used as templates for reverse transcription PCR (RT-PCR). The wtSYNV and rSYNV RT-PCR products (∼1500 bp) were digested with *Bsm*BI or *Apa*I and analyzed on 1.5% agarose gels. Positions of DNA size markers (M) are shown in base pairs.

To engineer rSYNV cDNA clones, the full-length SYNV gRNA (13.7-kilobase) was amplified by reverse transcription-PCR (RT-PCR), and the cDNAs were inserted into an *Agrobacterium* binary expression vector to produce pSYNV for transcription of agRNAs in agroinfiltrated leaves (Fig 1A). The SYNV cDNA was positioned between a truncated cauliflower mosaic virus (CaMV) double 35S promoter (2X35S) and a self-cleaving hepatitis delta virus (HDV) ribozyme sequence to ensure synthesis of SYNV agRNA transcripts with exact 5′- and 3′-ends (Fig 1A). To reconstitute infectious NCs *in vivo*, a mixture of *Agrobacterium* cultures harboring the pSYNV, and the pGD-N, pGD-P and pGD-L supporting plasmids that encode the N, P and L core proteins needed for NC formation with the SYNV agRNA, were co-infiltrated into *N*. *benthamiana* leaves. The mixture also contained *Agrobacteria* harboring the tomato bushy stunt virus (TBSV) p19, barley stripe mosaic virus (BSMV) γb and tobacco etch virus (TEV) P1/HC-Pro VSRs to minimize host RNA silencing responses [26,27], as this strategy has proven to be successful for *in vivo* reconstitution of an SYNV-derived minireplicon (MR) [26]. Approximately 20 days post infiltration (dpi), a small percentage of the infiltrated plants (∼5%) developed typical systemic SYNV symptoms such as stunting, leaf cupping and vein clearing (Fig 1B and Table 1). Immunoblotting with antibodies raised against SYNV virions revealed comparable amounts of the G, N, M and P proteins in the rSYNV- and wild-type SYNV (wtSYNV)-infected tissues (Fig 1C). As predicted from our previous SYNV MR experiments showing that fluorescent reporter expression requires the core proteins and is greatly enhanced by co-expression of VSR proteins [26], plants agroinfiltrated with mixtures lacking any of the core protein plasmids failed to develop symptoms, as was also the case with 145 plants infiltrated with mixtures lacking the VSR plasmids (Table 1).

**Table 1:** Systemic infection rates of SYNV antigenomic cDNA and derivatives in *Nicotiana benthamiana* supported by various combinations of core proteins and viral suppressors of RNA silencing (VSRs).

Antigenome derivatives^a^	Core proteins^b^	VSRs^c^	Systemic infection (No. of infected/inoculated plants)			
			Exp. 1	Exp. 2	Exp. 3	Total (percentage)
SYNV	N+P+L	-	0/40	0/60	0/45	0/145 (0%)
SYNV	N+P+L	+	2/40	3/60	5/90	10/190 (5.3%)
SYNV	N+P	+	0/40	0/60	0/45	0/145 (0%)
SYNV	N+L	+	0/40	-	-	0/40 (0%)
SYNV	P+L	+	0/40	-	-	0/40 (0%)
SYNV	NPL	+	3/40	11/60	4/45	18/145 (12.4%)
SYNV	NPL+L	+	11/40	9/40	13/45	33/125 (26.4%)
SYNV-GFP	NPL+L	+	12/60	10/40	10/45	32/145 (22.1%)
SYNV-GFP-ΔG	NPL+L	+	2/60	3/90	2/90	7/240 (2.9%)
SYNV-GFP-Δsc4	NPL+L	+	0/60	0/60	0/45	0/165 (0%)
SYNV-GFP-ΔM	NPL+L	+	0/60	0/60	0/45	0/165 (0%)
SYNV-GFP-Δsc4 + MR-sc4-RFP	NPL+L	+	0/60	-	-	0/60 (0%)

Equal volumes of *Agrobacterium* cultures carrying the plasmids encoding each of the antigenome derivatives, core proteins and VSRs were mixed and infiltrated into *N*. *benthamiana* leaves. Systemically infected plants were confirmed by symptom inspection and RT-PCR. The number of systemically infected per inoculated plants in three independent experiments (Exp.) were used to calculate the total percentage of systemic infectivity.

^a^ SYNV antigenomic cDNA and its derivatives were cloned into the pCB301 binary vector. MR-sc4-RFP is an SYNV antigenome minireplicon derivative containing an sc4 gene substitution for the N ORF and a RFP gene substitution for the P ORF.

^b^ N: pGD-N; P: pGD-P; L: pGD-L; NPL: pGD-NPL containing N, P and L tandem expression cassettes.

^c^ VSRs: pGD vectors expressing BSMV γb, TBSV p19 and TEV P1/HC-Pro, respectively.

Mechanical transmission assays showed that rSYNV is highly sap-transmissible (up to 100% of inoculated plants), and that the rSYNV and wtSYNV strains elicited indistinguishable disease symptoms on upper emerging leaves starting from ~13 dpi (S1A Fig). Moreover, as observed in previous studies [7,25], transmission electron microscopy revealed similar cytopathological effects in wtSYNV- and rSYNV-infected cells, which contained large numbers of bacilliform particles in perinuclear spaces around the periphery of the nuclei (S1C Fig).

During plasmid construction, we observed that the pSYNV cDNA contained a mutation at nucleotide (nt) 13,592 in the *L* gene sequence that changed a Lys codon (AAA) in the wtSYNV strain to an Arg (AGA) codon and created a B*sm*BI restriction site that could be used as a genetic marker for rSYNV. Therefore, to verify that the agroinoculated plants contained rSYNV rather than wtSYNV contaminants, RNA was extracted and an ~1,500 nt cDNA encompassing the mutant sequence was amplified by RT-PCR and digested with B*sm*BI. As expected, the cDNAs from rSYNV-infected tissue produced ~500 and 1,000 nt bands, but the wtSYNV cDNA was not digested, whereas a control digestion at an adjacent *Apa*I site provided identical digestion patterns with both cDNAs (Fig 1D). Moreover, the B*sm*BI restriction site mutation was stably maintained in the progeny genomes of rSYNV after mechanical transmission with leaf sap extracted from agroinfected plants (S1B Fig). These results demonstrate conclusively that rSYNV was derived from the cloned plasmids.

### Optimization of core protein expression improves the efficiency of SYNV recovery

Rescue of recombinant NSR viruses from cDNA is generally inefficient because multiple plasmids must be simultaneously introduced into single cells to reconstitute infectious virus [13,14,18–20]. Therefore, we sought to improve the recovery of rSYNV by reducing the numbers of plasmids delivered by agroinfiltration. We cloned the N, P and L gene expression cassettes into the pGD vector to develop a single multi-expression plasmid designated pGD-NPL (See S1 Protocols for cloning details). This strategy reduces the *Agrobacterium* strains required for expression of the N, P and L proteins from three to one, while also ensuring simultaneous expression of the three core proteins in a given cell. When the pGD-NPL bacterial culture was substituted for the mixture containing the pGD-N, pGD-P and pGD-L plasmids (N+P+L mixture), along with *Agrobacterium* strains harboring pSYNV and the VSRs, the proportion of infected plants increased more than two-fold compared with the N+P+L mixture, and resulted in ~12% of the agroinfiltrated plants developing systemic infections (Table 1).

**Fig 2:**
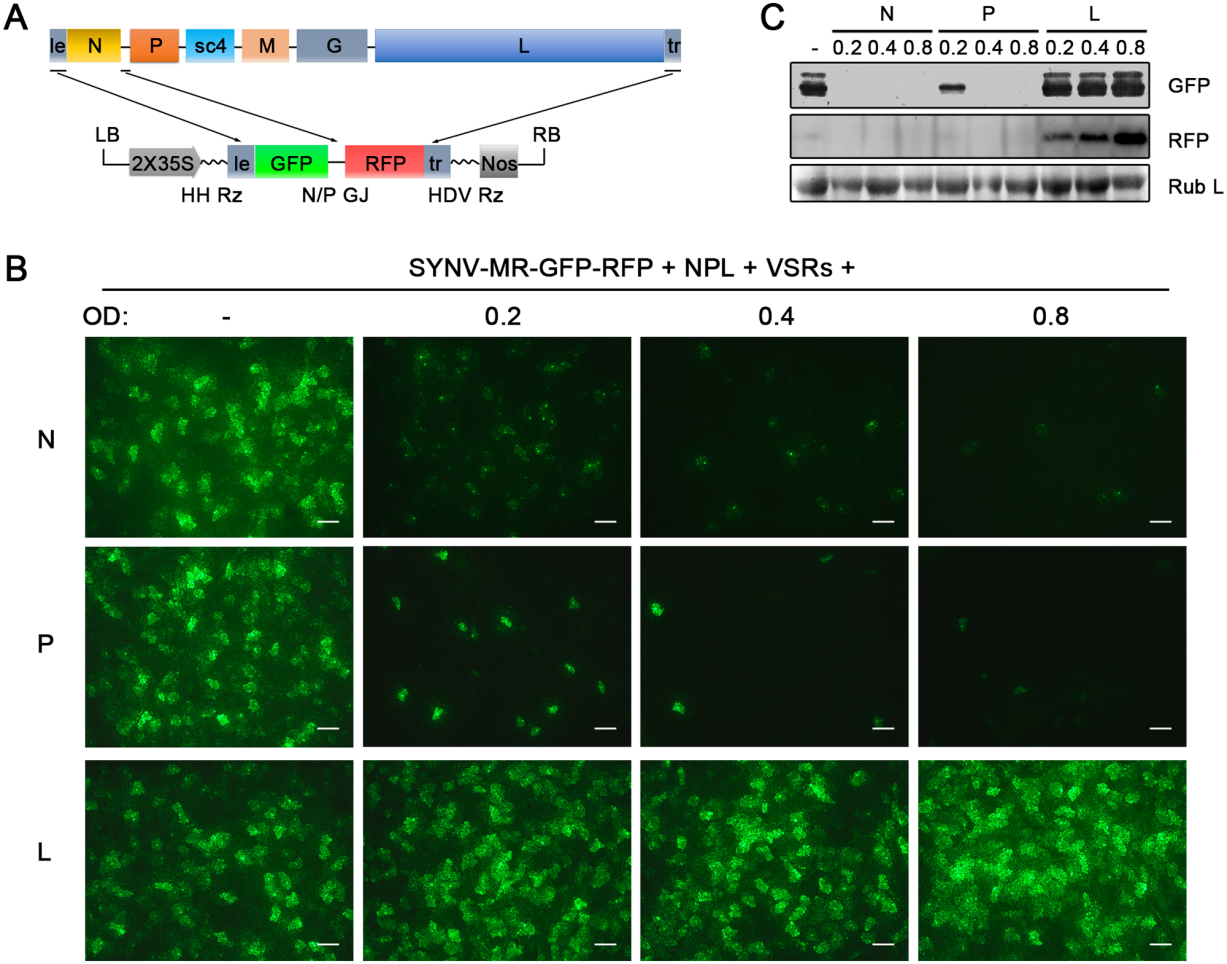
Improvement of SYNV minireplicon (MR) expression by optimizing the core protein ratios. (A) Schematic representation of the SYNV MR-GFP-RFP containing GFP and RFP reporter genes substituted for the N and P ORFs. The SYNV MR-GFP-RFP antigenomic RNA was transcribed from a CaMV double 35S promoter (2X35S), and flanked by a Hammerhead ribozyme (HH Rz) and HDV ribozyme (HDV Rz) sequence to produce exact 5′- and 3′- ends. le: leader; tr: trailer; LB: left border sequence; RB: right border sequence; Nos: nopaline synthase terminator; N/P GJ: N/P gene junction. (B) Visualization of plaques expressing GFP reporter protein in infiltrated plants. Equal volumes of 0.8 OD *Agrobacterium* cultures harboring the SYNV MR-GFP-RFP, pGD-NPL and the three VSRs plasmids were mixed and infiltrated into *N*. *benthamiana* leaves. Additional volumes of bacterial cultures containing the pGD-N (upper panels), pGD-P (middle panels) or pGD-L plasmids (bottom panels) at 0.2, 0.4 or 0.8 OD as indicated on the top of panels, were also included in the mixture to test their effects on reporter expression. Infiltrated leaves were photographed at 9 dpi with a fluorescence microscope under the GFP channel. Scale bar, 200 μm. (c) Detection of GFP and RFP protein levels in agroinfiltrated leaves by Western blotting using GFP- and RFP-specific antibodies. The Coomassie blue-stained Rubisco large subunit (Rub L) serves as a total protein loading control.

The relative ratios of the supporting N, P and L proteins are also important for recovery of recombinant NSR viruses [18]. Therefore, we used our previously developed SYNV MR fluorescent reporter expression assay [26] to determine recovery conditions that might lead to increased efficiency of rSYNV generation. In this assay, we constructed a SYNV MR derivative, which contains GFP and Red fluorescent protein (RFP) reporter genes substituted for the SYNV N and P ORFs, respectively, and the flanking 5′ le and 3′ tr sequences (Fig 2A) [26]. The MR plasmid, pSYNV-MR-GFP-RFP, when co-delivered with pGD-NPL and the VSR plasmids via agroinfiltration, exhibited intense GFP and RFP fluorescent foci throughout infiltrated regions (Fig 2B and S2 Fig). To optimize the ratio of core protein expression, different concentrations of *Agrobacterium* cultures harboring the pGD-N, pGD-P or pGD-L plasmids were added to the pGD-NPL culture. These mixtures were co-infiltrated into *N*. *benthamiana* leaves, and the appearance of GFP and RFP fluorescent foci was observed by fluorescence microscopy (Fig 2B and S2 Fig). To our surprise, addition of extra amounts of *Agrobacterium* containing the N or P plasmids greatly reduced the expression of the GFP and RFP reporter genes, whereas supplying additional L plasmid (NPL+L mixture) increased the MR reporter foci in the infiltrated leaves (Fig 2B and S2 Fig). Note that the increasing amounts of N protein expression appeared to result in a strong reduction in the strength of reporter expressions, whereas additional P protein expression drastically reduced the numbers of fluorescent foci (Fig 2B and S2 Fig, N and P panels). These MR experiments suggested that higher rSYNV recoveries might be obtained if L protein expression was increased, and this proved to be the case when we added an extra volume of bacteria harboring the pGD-L plasmid to the NPL mixture. This mixture (NPL+L) led to systemic symptoms in ∼ 26% of the infiltrated plants, compared with ~ 12% with the NPL mixture, and ~ 5% with the N+P+L mixture (Table 1). Thus, reducing the numbers of supporting plasmids while increasing the abundance of the L plasmid dramatically improved rSYNV recovery.

### A rSYNV vector exhibits high levels of stable GFP expression

**Fig 3:**
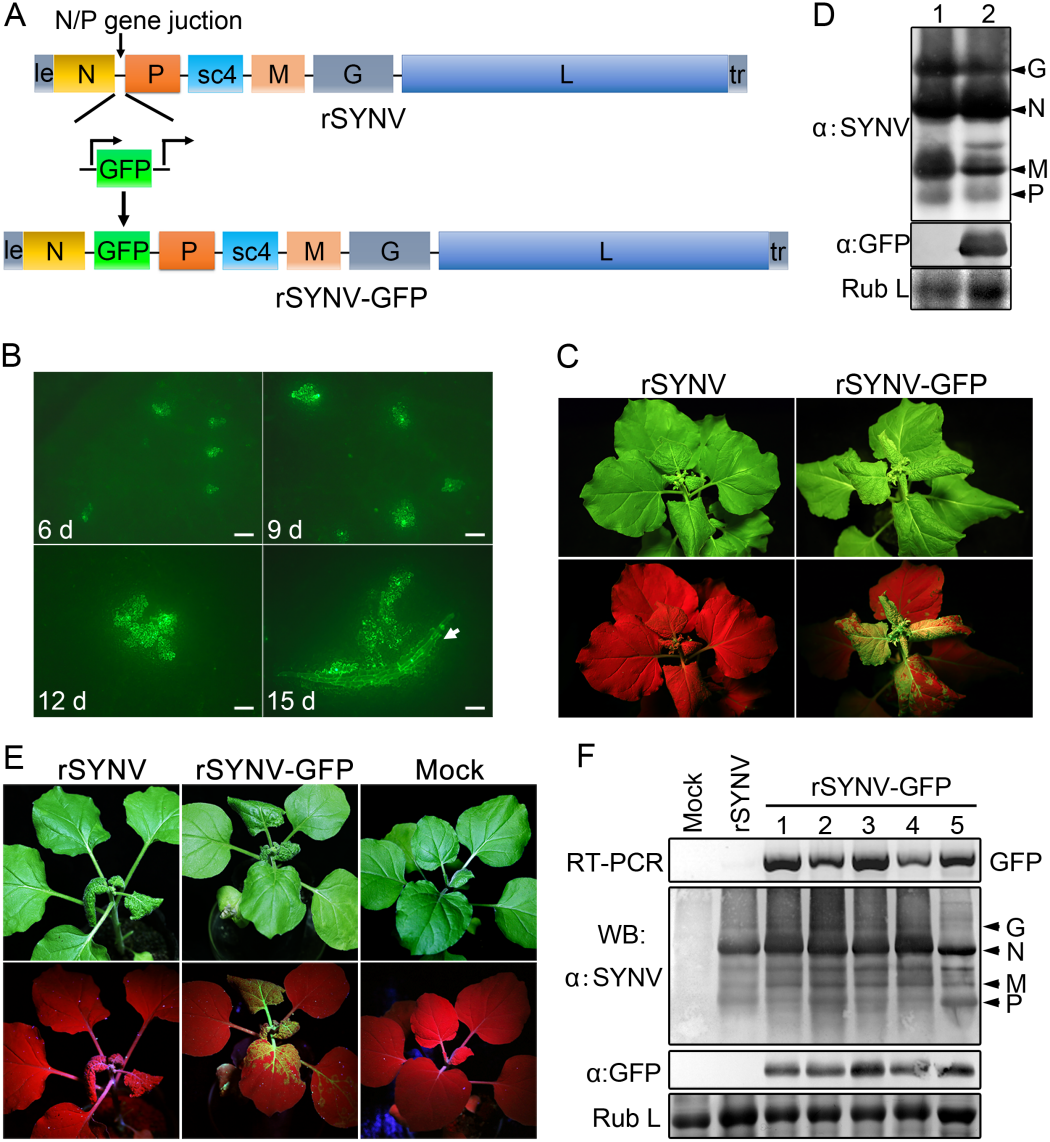
Expression of GFP engineered into rSYNV. (A) Diagram showing insertion of the GFP gene between the N and P genes of the rSYNV antigenome to produce rSYNV-GFP. The recombinant vector transcribes GFP mRNA under the control of a duplicated N/P gene junction sequence. (B) Time course of GFP foci appearing in *N*. *benthamiana* leaves after agroinfiltration with rSYNV-GFP derivatives. *N*. *benthamiana* leaves were agroinfiltrated with bacterial mixtures containing plasmids encoding the rSYNV-GFP agRNA, the N, P, L proteins and the VSR suppressor proteins, and photographed by fluorescence microscopy at 6, 9, 12 and 15 dpi. The white arrow indicates GFP entering a leaf vein. Scale bar, 200 μm. (C) Symptoms of rSYNV- and rSYNV-GFP-infected plants. Photographs were taken at 30 dpi under white light (upper panels) and ultraviolet (UV) light (bottom panels). (D) Immunoblot analysis of SYNV proteins and GFP expression in plants systemically infected with rSYNV-GFP. Total proteins extracted from rSYNV (lane 1) and rSYNV-GFP (lane 2) infected plants were analyzed by Western blotting with antibodies against disrupted SYNV virions (α:SYNV) or GFP (α:GFP). (E) Stable maintenance of GFP during serial passages of rSYNV-GFP in *N*. *benthamiana*. rSYNV-GFP passaged to healthy plants by mechanical inoculation, and after 5 serial passages, the infected plants were photographed at 16 dpi under white (upper panels) and UV light (bottom panels). (F) Total RNAs and proteins were extracted from upper infected leaves of each plant passage and analyzed by RT-PCR using GFP-specific primers or by immmunoblotting (WB) with αSYNV and αGFP antibodies. Numbers 1 to 5 on the top of the panel represent passages No. 1 to 5, respectively. Coomassie blue stained Rubisco large subunit (Rub L) was used as a loading control.

To develop an rSYNV vector for foreign gene expression in plants, a duplicated N/P gene junction sequence along with the GFP coding sequence was inserted into the pSYNV plasmid between the N and P genes to generate pSYNV-GFP (Fig 3A). In this configuration, GFP mRNA synthesis is initiated immediately after termination of the upstream N protein mRNA synthesis by the duplicated N/P gene junction, and is followed by P mRNA synthesis that is directed by the native N/P gene junction. The pSYNV-GFP plasmid was agroinfiltrated into *N*. *benthamiana* leaves along with the bacterial mixture harboring the NPL+L and VSR plasmids. At about 6 dpi, GFP foci began to appear in single cells randomly distributed throughout the infiltrated tissue, and by 9 dpi fluorescence of these cells became more intense and faint fluorescence began to appear in surrounding adjacent cells (Fig 3B). By 12 dpi, fluorescence of the neighboring cells was clearly evident, and more extensive tissue fluorescence was obvious by 15 dpi, and in some cases, fluorescence was evident in isolated leaf veins (Fig 3B, Note white arrow). By ∼20 dpi, the newly emerging leaves of some infiltrated plants exhibited tight curling and yellow net symptoms typical of SYNV infections, and the rSYNV-GFP and rSYNV viruses appeared to be equally infectious based on the appearance of systemic symptoms in inoculated plants (Table 1). When monitored under long wavelength ultraviolent (UV) light, the symptoms in recombinant SYNV-GFP (rSYNV-GFP)-infected leaves were accompanied by strong GFP fluorescence (Fig 3C). Western blot analyses also revealed similar levels of the G, N, M and P proteins in uninoculated upper leaves systemically infected by rSYNV and rSYNV-GFP, and showed that GFP expression was abundant in rSYNV-GFP-infected leaves, but was absent in rSYNV-infected leaves (Fig 3D).

With rare exceptions, plant positive-strand RNA vectors are unable to maintain foreign genes stably during plant-to-plant transfers [28,29]. To investigate the stability of rSYNV-GFP, healthy plants were mechanically inoculated with rSYNV-GFP sap preparations and GFP expression was monitored under UV light and by Western blot analysis. Intense GFP fluorescence could be detected for at least five rSYNV-GFP serial passages (Fig 3E) and GFP protein was expressed at similar levels in all plant passages (Fig 3F). RT-PCR analysis with GFP specific primers also indicated that the GFP insert was stably maintained in the progeny virus genomes (Fig 3F). These results demonstrate that rSYNV can be engineered for stable expression of foreign genes.

### The sc4 protein and nucleocapsids are involved in SYNV cell-to-cell movement

**Fig 4:**
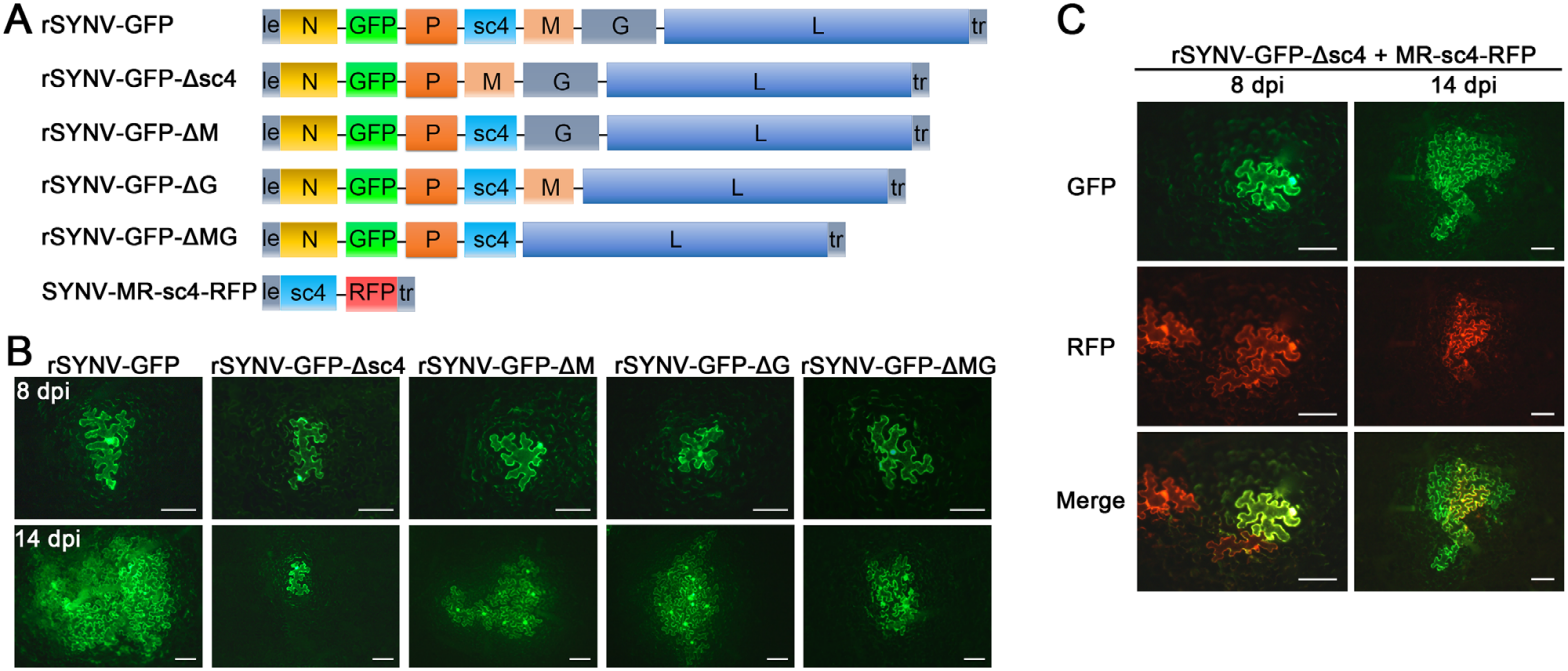
Cell-to-cell movement analysis of rSYNV sc4, M and G deletion mutants. (A) Schematic representation of rSYNV-GFP and rSYNV-GFP mutants with Δsc4, ΔM or ΔG deletions. Note that the SYNV gene order is shown in the antigenome sense. (B) Cell-to-cell movement of rSYNV-GFP and deletion derivatives. *N*. *benthamiana* leaves were agroinfiltrated with the rSYNV-GFP plasmid or the indicated mutant rSYNV-GFP plasmids along with supporting plasmids for expression of the N, P, L core proteins and the VSRs. Leaves were photographed with a fluorescence microscope at 8 and 14 dpi. Scale bar, 100 μm. (C) Complementation of rSYNV-eGFP-Δsc4 cell-to-cell movement by the sc4 protein expressed *in trans* from the MR-sc4-RFP minireplicon. *Agrobacterium* strains harboring the rSYNV-eGFP-Δsc4 plasmid, the MR-sc4-RFP plasmid, along with the supporting plasmids indicated in the panel B legend, were mixed and infiltrated into *N*. *benthamiana* leaves. Fluorescence images for GFP, RFP and the overlaid images are shown at 8 and 14 dpi. Scale bar, 100 μm.

Having tagged rSYNV with the GFP reporter, we carried out experiments to investigate the requirements of the sc4, M and G genes for SYNV cell-to-cell movement because these genes appear not to be required for virus replication [7,26]. To knockout the sc4, M or G genes in rSYNV-GFP, the entire transcription unit of a given gene was deleted (Fig 4A), beginning with the upstream transcription start site through the entire ORF and the downstream transcription termination sequence [7]. Bacteria harboring the sc4 (rSYNV-GFP-Δsc4), M (rSYNV-GFP-ΔM) and G (rSYNV-GFP-ΔG) deletion mutants were each agroinfiltrated into *N*. *benthamiana* leaves along with the NPL+L and the VSR bacterial mixture, and their movement patterns were compared with those of rSYNV-GFP. In rSYNV-GFP infiltrated leaves, discrete GFP fluorescent foci were first seen in single cells at about 6 to 8 dpi and spread into adjacent cells extensively by 14 dpi (Fig 4B). However, the rSYNV-GFP-Δsc4 foci were restricted to single cells at both 8 and 14 dpi (Fig 4B). These data demonstrate that the sc4 protein has an essential role in viral cell-to-cell movement and possesses the characteristics of a virus MP. In contrast, both the rSYNV-GFP-ΔM and rSYNV-GFP-ΔG mutants were capable of local movement, albeit less efficiently than rSYNV-GFP (Fig 4B). In addition, an M and G double mutant, rSYNV-GFP-ΔMG, was still able to move from cell-to-cell, although the rates of movement appeared to be lower than those of the rSYNV-GFP-ΔM and rSYNV-GFP-ΔG mutants (Fig 4B).

Because mRNA transcription of nonsegmented NSR viruses progressively attenuates at each gene junction site [30], deletion of a given transcription unit may lead to alteration of viral mRNA ratios that could result in virus attenuation [31]. Therefore, we generated a second set of mutants, in which the RFP gene was substituted for the sc4, M or G genes respectively (S3 Fig), and assessed these mutants for their localized movement abilities. Again, only the sc4 substitution mutant (rSYNV-GFP-Δsc4:RFP) was unable to initiate cell-to-cell movement (S3B Fig). Thus, these two sets of data collectively show that both the M and G proteins are dispensable for cell-to-cell movement and suggest that uncoiled NCs are able to function in localized movement.

To further confirm that the sc4 protein, but not the RNA sequence is required for local movement, we tested whether or not the sc4 protein expressed *in trans* can complement rSYNV-GFP-Δsc4 cell-to-cell movement. Because SYNV cell-to-cell movement becomes evident only after about 9 dpi (Figs 3B and 4), to synchronize sc4 expression with critical steps in SYNV movement, we took advantage of SYNV MR-directed expression of proteins, which has been shown to persist for up to 20 days [26]. To this end, we constructed SYNV MR-sc4-RFP (Fig 4A), which substitutes the sc4 ORF for the N ORF and the RFP ORF for the P ORF (See S1 Protocols for cloning details), and evaluated whether persistent expression of sc4 could facilitate rSYNV-GFP-Δsc4 movement *in trans*. As shown in Fig 4C, the MR-sc4-RFP infiltrated regions exhibited RFP fluorescence in some cells at 8 dpi, which provided a marker for MR-mediated gene expression. In some instances, GFP fluorescence produced by rSYNV-GFP-Δsc4 was observed in single cells that also showed RFP fluorescence (Fig 4C, left panels), indicating that those cells had received all plasmids necessary for MR-sc4-RFP and rSYNV- GFP-Δsc4 reconstitution and expression. In these cases, GFP fluorescence continued to increase in cells and spread beyond the RFP fluorescence by 14 dpi, which mostly remained confined to single cells or to a very limited number of cells (Fig 4C, right panels). These results suggest that MR-sc4-RFP did not invade adjacent cells extensively, but that the sc4 protein produced *in trans* was able to expedite limited cell-to-cell transit of the rSYNV-GFP-Δsc4 reporter virus.

### The glycoprotein is dispensable for systemic SYNV infection but is required for morphogenesis

**Fig 5:**
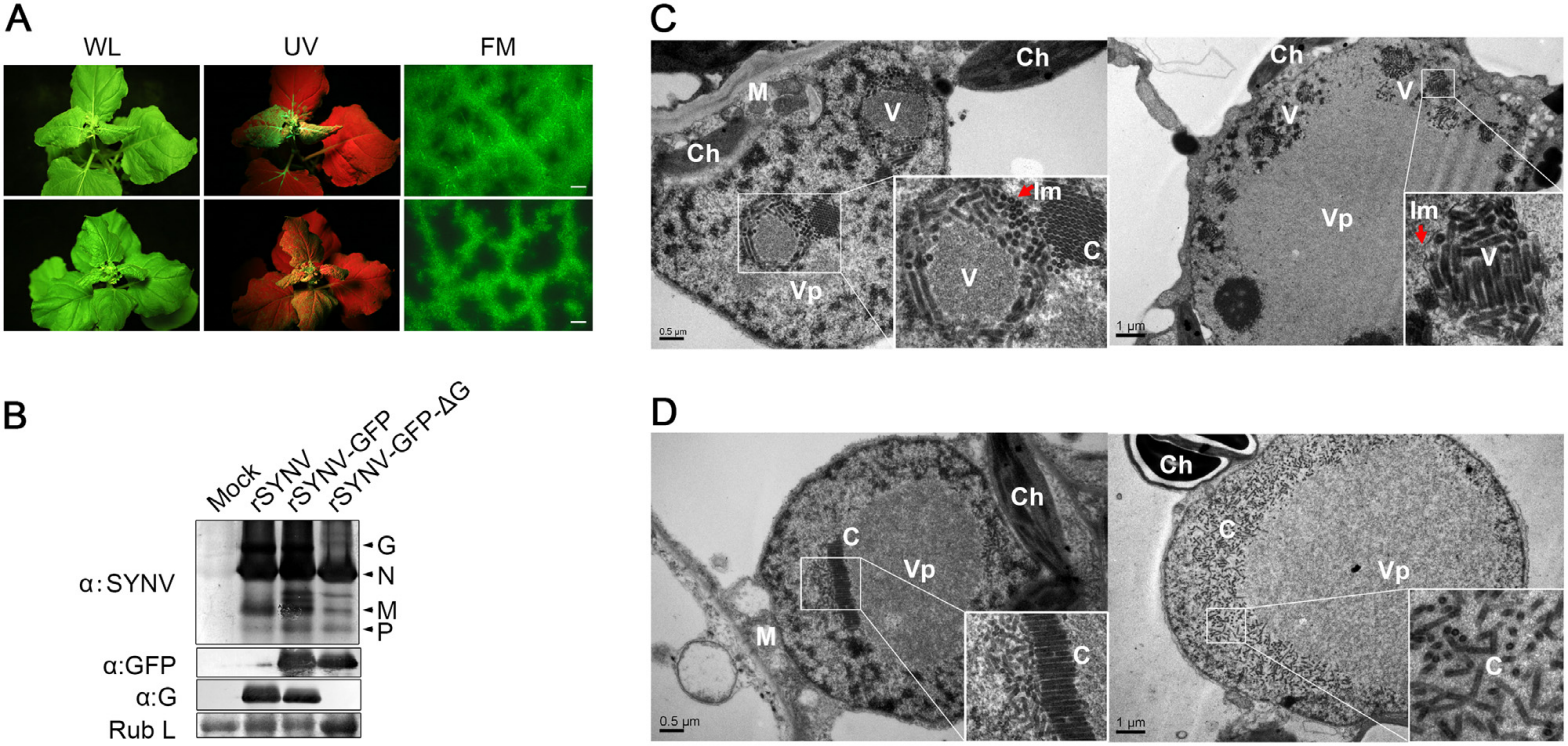
Role of the SYNV glycoprotein in systemic infection and virion maturation. (A) Symptoms of *N*. *benthamiana* plants after agroinfiltration with rSYNV-GFP and rSYNV-GFP-ΔG infectious clones. Leaves were photographed under white light (WL) or UV light at 30 dpi for rSYNV-GFP (upper panels) and at 35 dpi for rSYNV-GFP-ΔG (lower panels). Leaf tissue was also photographed with a fluorescence microscope (FM) to show GFP expression patterns in the mesophyll tissue. Scale bar, 200 μm. (B) Total proteins extracted from newly emerged leaf tissues were detected by immunoblotting with antibodies raised against SYNV virions (αSYNV), G protein (αG) or GFP (αGFP). The bottom panel is a loading control showing the Coomassie blue stained Rubisco large subunit (Rub L). (C and D) Electron micrographs of thin sections of *N*. *benthamiana* plants infected with rSYNV-GFP (C) and rSYNV-GFP-ΔG (D). The boxed sectors in the panels are magnified (40,000 X) to highlight the appearance of the virions and nucleocapsid cores. The red arrows in (C) show cross sections of the invaginated inner nuclear envelopes surrounding the enveloped virions. V: virion; C: core; Vp: viroplasm; Im: inner nuclear membranes; Ch: chloroplast; M: mitochondria.

To determine the viral proteins involved in systemic infection, the agroinfiltrated plants shown in Fig 4 were monitored for appearance of symptoms and GFP fluorescence in upper uninoculated leaves. The rSYNV-GFP-ΔG mutant was able to develop systemic infections and induced symptoms similar to rSYNV-GFP (Fig 5A), although the proportion of systemically infected plants was drastically reduced compared with the rSYNV-GFP inoculations (∼3% systemically infected plants for rSYNV-GFP-ΔG as compared to ∼22% for rSYNV-GFP infiltrations, Table 1). In rSYNV-GFP-ΔG infections, fluorescence first appeared in the upper leaves at ∼25 dpi and began to spread from the leaf veins, but was mostly confined to the upper leaf veins even at 35 dpi (Fig 5A, lower panels). In contrast, the rSYNV-GFP-infected upper leaves exhibited GFP fluorescence throughout the mesophyll tissues by 30 dpi (Fig 5A, upper panels). Nevertheless, Western blot analyses revealed only moderately reduced abundances of the SYNV N, M, and P proteins and GFP in the systemically infected leaves when compared with those of the rSYNV-GFP infections, and confirmed the absence of G protein in the systemic leaves infected by rSYNV-GFP-ΔG (Fig 5B). These results show that the SYNV G protein is not required for symptom development and systemic movement, but may facilitate infection by unknown mechanism(s). As expected from its inability in cell-to-cell movement, the rSYNV- GFP-Δsc4 mutant was defective in systemic infection (Table 1). Interestingly, although rSYNV-GFP-ΔM was capable of localized movement in the infiltrated leaves (Fig 4B), the mutant was unable to invade upper leaves as judged by visual inspection and RT-PCR analysis (Table 1). Hence, the M protein appears to be required for SYNV movement from primary infection foci into vasculature.

To investigate the roles of the G protein in SYNV morphogenesis and cytopathology, upper leaf tissues infected with rSYNV-GFP and rSYNV-GFP-ΔG were compared by transmission electron microscopy. As with previous studies of SYNV-infected plants and protoplasts [7,25], enlarged nuclei of rSYNV-GFP-infected cells contained large numbers of intact bacilliform particles (71.0 ± 3.4 nm diameter; n = 26) surrounded by invaginated inner nuclear envelopes (Fig 5C, as indicated by red arrows). Smaller nonenveloped bullet-shaped aggregates (53.0 ± 2.2 nm diameter; n = 26) were also observed in electron dense regions characteristic of the subnuclear viroplasms (Fig 5C, left panel). The larger enveloped particles have the appearance of mature enveloped virions, whereas the smaller nonenveloped particles appear to represent naked cores that have not yet completed morphogenesis [25]. In marked contrast, rSYNV-GFP-ΔG-infected cells contained only nonenveloped particles (54.3 ± 2.2 nm in diameter, n = 12) that were randomly distributed or occurred as orderly aligned arrays within the viroplasms (Fig 5D). Our data thus demonstrate that the G protein is required for morphogenesis of enveloped SYNV particles, and that in the absence of the G protein large numbers of NC cores accumulate in or near the viroplasms.

## Discussion

Recovery of infectious NSR viruses from cloned cDNAs for reverse genetic analyses is now routine for all animal NSR virus families. Although the procedures are quite inefficient, with 10^4^ to 10^7^ transfected cells per primary infected cell, recombinant virus particles released from primary infected cells can be passaged to permissive cell lines to obtain progeny viruses suitable for a variety of purposes [13,14,18–20]. Unfortunately, only a few insect vector cell cultures suitable for rescue of recombinant plant NSR viruses have been established [32,33]. Even these lines are difficult to maintain and to our knowledge, plasmids suitable for transient expression of multiple genes in these lines are unavailable. Moreover, introduction of multiple components into single plant cells after removal of the cell wall is inefficient and protoplast recoveries after transformation or viral transfection is low. In addition, transformation and high level expression of multiple viral proteins and RNAs in plant leaves is difficult due to the presence of the cell wall and the existence of potent plant antiviral gene silencing mechanisms [34,35]. Hence, to circumvent these problems, we turned to infiltration of *N*. *benthamiana* leaves with *Agrobacterium* strains harboring plasmids encoding the SYNV agRNAs and the N, P and L core proteins needed for *de novo* NC assembly, coupled with the use of VSRs proteins to suppress host RNA silencing. This approach has enabled *in planta* rescue of rSYNV from cDNAs with an infection phenotype identical to wtSYNV (Fig 1 and S1 Fig).

The recovery of rSYNV from agroinfiltrated plants was initially inefficient, as only ~5% of the agroinfiltrated plants developed systemic symptoms, but we were able to improve rSYNV recovery to ~26% of the infiltrated plants by optimizing the infection mixture components (Table 1). It is worth noting that the SYNV MR reporter system that we developed earlier [26] was invaluable in devising steps to rescue full-length rSYNV and to improve recovery efficiency. The MR derivatives provided a rapid assay to determine the functionality of agRNA derivatives and the optimum conditions for expression of the SYNV core components that could be applied to improve the efficiency of rSYNV recoveries in agroinfiltrated leaves (Fig 2). Similar MR systems have also proven to be very helpful for recovery of animal NSR viruses [8, 20], and we believe that time invested to develop MR derivatives will be very worthwhile in future studies to develop and optimize engineering of recombinant plant NSR viruses.

Although the general principles used for recovery of rSYNV are similar to those used for recoveries of NSR animal viruses, our study reveals several distinct aspects that merit consideration when developing strategies for generation of other recombinant plant NSR viruses. First, co-expression of VSRs to suppress potent RNA silencing response in plants proved to be extremely important for generation of rSYNV (Table 1). Similar strategies have previously been shown to be important for cDNA recoveries of complex plant positive-strand RNA viruses [36,37]. It is known that host gene silencing mechanisms generally reduce *Agrobacterium* transient gene expression [34], and that co-expression of VSRs can alleviate this limitation [35]. These VSR proteins most likely facilitate rSYNV recovery by reducing degradation of SYNV mRNAs and agRNA transcripts to permit high levels of N, P and L protein expression needed for efficient NC generation. In addition, VSR proteins may also prevent host antiviral RNA silencing machineries from degrading rescued virus and promote efficient virus spread during the initial stages of infection [38,39]. Second, our studies revealed a requirement of higher amounts of the L plasmid relative to the N and P plasmids for efficient SYNV recovery. The requirement for higher levels of L protein is counter-intuitive, because the abundance of the N and P proteins is much higher than the L proteins in nonsegmented NSR nucleocapsids [8,9]. Moreover, studies with NSR animal viruses have usually shown that high levels of N protein expression are correlated with more efficient rescues [18]. However, the relative molar ratios of N, P and L protein expressed in the agroinfiltrated leaves were not determined in our study due to low titers of L protein antibody, so it is possible that transient expression of the L protein (~242 KDa) is less efficient than those of the smaller N and P proteins (54 and 34 KDa, respectively). Third, unlike most NSR animal virus rescue systems, in which phage T7 RNA polymerase or host RNA polymerase I were used to direct intracellular transcription of viral agRNAs [18–20], we used a CaMV 35S promoter, which relies on the endogenous RNA polymerase II machinery, to drive the SYNV agRNA and core protein expression. The 35S promoter was truncated to permit exact 5′ initiation of SYNV agRNA transcription, and this strategy circumvents the use of hammerhead ribozyme cleavage to generate authentic 5′ ends of agRNA transcripts [26]. Since the 35S promoter is known to function in various dicots and monocots species, it is likely that the plasmid-based system developed in the present study will be applicable to other families of plant NSR viruses.

We have engineered an SYNV reporter virus (rSYNV-GFP) that expresses GFP stably even after repeated mechanical passages (Fig 3). We have used this reporter virus to provide a simple visual assay to follow local and systemic spread of rSYNV, and to investigate the genetic requirements for SYNV movement. As with other plant viruses, NSR viruses must move from initially infected cells to neighboring cells through MP-gated plasmodesmata [3]. Studies based mainly on positive-strand RNA viruses have proposed two major mechanisms whereby plant viruses move from cell-to-cell. These mechanisms involve either direct interactions of MPs with either viral genomes or with intact virions, which then move through MP modified plasmodesmata [40,41]. However, direct evidence has not previously been available as to the nature of the plant NSR virus infectious entities that navigate intercellular connections. In the case of plant rhabdoviruses, previous indirect studies have suggested that sc4, and similar plant rhabdovirus homologs exhibit MP properties [7,42–44]. The failure of the rSYNV-GFP-Δsc4 mutant to move from cell-to-cell (Fig 4 and S3 Fig) now provides the first direct evidence that sc4 is the SYNV MP and supports a movement function for sc4 homologs of other plant rhabdoviruses. However, in contrast to the sc4 deletion mutant, the M and G single or double deletion mutants are capable of localized movement (Fig 4 and S3 Fig). These findings argue against previous speculations that the M protein functions as an essential component of cell-to-cell movement complexes or that mature virions move through ER tubules and desmotubules into adjacent cells [45,46]. Rather, our data suggest a model whereby a portion of the NCs in or adjacent to the viroplasms interact with the sc4 protein and are exported from the nucleus. Consistent with this model, the putative MPs of several plant NSR viruses, i.e. the P3 protein of rice yellow stunt nucleorhabdovirus and the NSm proteins of several tospoviruses in the *Bunyaviridae* family, have been shown to bind directly to the their cognate N proteins [42,47–49], although similar interactions have not been reported for the SYNV sc4 and N proteins.

Mature virions of enveloped NSR viruses are formed by a budding process, during which the M proteins function to condense and coil the NCs into cores that then bud through host membranes to acquire phospholipid envelopes and the glycoprotein spikes [50,51]. The budding process is essential for release of animal viruses from infected cells, while the surface glycoproteins have critical roles during cellular entry [50,52]. Although such processes presumably occur during insect vector infections of plant enveloped NSR viruses, the functions of the glycoprotein during plant host infections have remained obscure. Our deletion analyses now show that the rSYNV-GFP-ΔG mutant is able to cause systemic infection in plants and induce typical symptoms (Fig 5A). However, the ΔG mutant failed to undergo morphogenesis, resulting in large numbers of naked cores accumulating in the viroplasms (Fig 5D). Such cytopathic structures are reminiscent of the striking arrays of cores present in the nuclei of SYNV-infected protoplasts treated with tunicamycin [25], an inhibitor that blocks G protein N-glycosylation [53]. These data are also consistent with previous findings by Sin et al [54], who have used a reassortment-based forward genetics approach to map the insect transmissibility determinant of tomato spotted wilt virus (TSWV), a tripartite NSR virus in the *Bunyaviridae* family. Sin et al. have shown that several TSWV single-lesion isolates with mutations in the glycoprotein precursor are defective in virion assembly and insect transmissibility, but are able to infect plants. Hence, the glycoproteins and the mature virions of two distinct plant NSR virus families are dispensable for systemic infection of plants. Interestingly, the SYNV M deletion mutant was unable to invade upper leaves (Table 1), although its local movement appeared to be as efficient as the rSYNV-GFP-ΔG (Fig 4B). These results suggest that the M protein may play a role in long-distance movement, perhaps by promoting efficient NC coiling [7,51] and/or NC entry into the vasculature.

In conclusion, we have developed a plasmid-based reverse genetics system for recovery of rSYNV directly in agroinfiltrated plants. This achievement permits investigation of fundamental aspects of plant rhabdovirus biology and pathology that were technically unapproachable previously. We anticipate that similar approaches can be applied to other plant NSR viruses for refined studies of plant infections and insect interactions. Given the exceptional stability of the GFP protein during plant-to-plant transmission of rSYNV-GFP (Fig 3E and 3F), and the widespread use of recombinant animal rhabdoviruses as vectors for expression of antigens and delivery of therapeutic genes [22,23,55], rSYNV and other plant NSR viruses also hold great promise for biotechnological applications.

## Methods

### Construction of SYNV infectious clones

Total RNA extracted from SYNV-infected *N*. *benthamiana* plants was used for RT-PCR amplification of the full-length cDNA of agRNA with a high-fidelity KOD-Plus-Neo DNA polymerase (Toyobo, Osaka, Japan) and the forward 5′-tttcatttggagaggAGAGACAGAAACTCAGAAAATACAAT-3′ and reverse 5′-atgccatgccgacccAGAGACAAAAGCTCAGAACAATCCCTAT-3′ primers. The forward primer contains 15-nt overhangs (lowercase letters) complementary to the 3′ end of the 35S promoter, whereas the reverse primer contains 15-nt overhangs (lowercase letters) complementary to the 5′ end of the HDV ribozyme, respectively. To generate a binary plasmid for intracellular transcription of SYNV agRNA after agroinfiltration, the full-length cDNA was cloned into a modified pCB301-2X35S-Nos plasmid [56]. The pCB301-2X35S-Nos plasmid was linearized by S*tu*I and S*ma*I double digestion, which removes the sequence between the transcription initiation site of 35S promoter and the multiple cloning sites immediately before the HDV ribozyme sequence. The linearized plasmid was recovered and assembled precisely with the SYNV cDNA by using an In-Fusion HD PCR Cloning Kit (Clontech, Japan). The resulting plasmid (pSYNV) was sequenced to confirm its authenticity and correct assembly, and was expected to transcribe the full-length SYNV antigenome with the exact 5′ end by 35S promoter and the exact 3′ end processed by the HDV ribozyme cleavages. Supporting pGD plasmids [57] directing expression of the SYNV N, P, and L proteins, as well as the BSMV γb, TBSV p19, and TEV P1/HC-Pro have been described previously [26]. The constructions of other plasmid derivatives used in this study are detailed in the S1 Protocols.

### *Agrobacterium* infiltration

Recombinant binary plasmids were electroporated into *Agrobacterium tumefaciens* strain GV3101 and *N*. *benthamiana* agroinfiltrations were performed essentially as described [26]. Bacterial cell suspensions were activated by acetosyringone, adjusted to an optical density (OD) A_600_ of 0.8 and incubated for 2 to 4 hr at room temperature. Immediately before infiltration, equal volumes of *Agrobacterium* cultures harboring the pGD-N, pGD-P, pGD-L (N+P+L mixture), pSYNV (or the rSYNV deletion derivatives), were mixed with one volume of bacterial mixture containing the BSMV γb, TBSV p19, and TEV P1/HC-Pro plasmids, unless otherwise stated. In the case of the NPL mixture, one volume of pGD-NPL culture at 0.8 OD was used to substitute for the N+P+L mixture. For the NPL+L mixture, equal volumes of the NPL and the L *Agrobacterium* cultures were mixed at 0.8 OD.

### Mechanical inoculation

Mechanical transmissions were carried out as previously described [58]. Briefly, 2 g of young leaves from plants infected for approximately 20 days were ground in a chilled mortar containing 5 mL of freshly prepared cold (~4°C) inoculation buffer (5% sodium sulfite and 2% Celite). Two leaves of *N*. *benthamiana* plants at the 4~5 leaf-stage were gently rubbed by hand with the leaf extracts. The inoculated plants were placed in an insect-free growth chamber at ~25°C and 60% relative humidity under a 16 h light/8 h dark photoperiod.

### Identification of a unique restriction site in pSYNV

Total RNAs were extracted from upper leaves of wtSYNV- and rSYNV-infected plants with Trizol reagent (Invitrogen). RT-PCR was carried out using AMV Reverse Transcriptase XL (Takara, Japan) and Phusion High-Fidelity DNA Polymerase (NEB, Beverly, MA) with SYNV-specific primers SYNV/11008/F and SYNV/12503/R (S1 Table). The PCR products were digested with *Bsm*BI or *Apa*I in a 20-μl reaction mixture and separated in 1.5% agarose gels. PCR products amplified from sap-inoculated plants were also sequenced to confirm stable maintenance of the genetic tag.

### Immunoblotting

Total proteins were extracted from healthy or infected *N*. *benthamiana* leaves and evaluated by Western blotting. Proteins separated by SDS-PAGE were either stained with Coomassie blue or transferred to nitrocellulose membranes and probed with polyclonal antiserum specific to the disrupted SYNV virions [59], the SYNV G [60], or monoclonal antibodies against GFP and RFP (Abcam, Cambridge, UK).

### Fluorescence microscopy

*N*. *benthamiana* leaves were examined with a Zeiss SteREO Lumar. V12 epifluorescence microscope with filter set Lumar 38 (excitation 470/40; emission 525/50) and Lumar 31 (excitation 565/30; emission 620/60) for GFP and RFP, respectively. The data were processed with LSM software Zen 2009 (Carl Zeiss).

### Electron microscopy

Systemically infected leaf tissues were fixed in 2.5% glutaraldehyde and 1% osmium tetroxide in 100 mM phosphate buffer (pH 7.0) essentially as described by Kong et al [61] and then embedded in Epon 812 resin as described by the manufacturer (SPI-EM, Division of Structure Probe, West Chester, USA). Ultrathin sections (70 nm) were mounted on formvar-coated grids, and then stained with uranyl acetate for 10 min followed by lead citrate for 10 min. The stained sections were examined under a transmission electron microscope (TEM; H-7650, Hitachi, Japan) at 80 kV accelerating voltage.

## Supporting Information

S1 Protocols Descriptions of plasmid construction details. (DOCX)

S1 Table List of primers used in this study. (DOCX)

S1 Fig Indistinguishable biological characteristics of the wtSYNV and rSYNV. (A) Similar symptom phenotypes were observed at 25 dpi in *N*. *benthamiana* plants mechanically inoculated with wtSYNV and rSYNV sap preparations. The rSYNV sap was derived from symptomatic leaves of plants that had been infected by agroinfiltration with SYNV infectious clone. Mock: buffer inoculated plants. (B) Sequence comparison of rSYNV and wtSYNV cDNA regions encompassing the mutation site. Note: The rSYNV and wtSYNV sequences shown above the graphs are presented in the antigenomic sense, and the cytosine mutation (No.13,592) in rSYNV is shown in red. (C) Thin sections of infected plant cells are presented to show the similar cytopathology of cells infected with wtSYNV and rSYNV. In both sections, mature virions (V) were found within perinuclear spaces surrounded by the inner nuclear membrane (Im) and the outer nuclear membrane (Om). Nuc: nucleus; M: mitochondria. Scale bar, 0.2 μm.
(TIF)

S2 Fig Optimizing core protein ratios improved SYNV minireplicon (MR) RFP reporter protein expression. Equal volumes of *Agrobacterium* cultures at 0.8 OD harboring the SYNV MR-GFP-RFP, pGD-NPL and the three VSRs plasmids were mixed and infiltrated into *N*. *benthamiana* leaves. Additional volumes of bacterial cultures containing the pGD-N (upper panels), pGD-P (middle panels) or pGD-L plasmids (bottom panels) at 0.2, 0.4 or 0.8 OD as indicated on the top of panels, were also included in the mixture to test their effects on reporter expression. Infiltrated plants were photographed at 9 dpi with a fluorescence microscope under RFP channel. Scale bar, 200 μm.
(TIF)

S3 Fig Requirements of the sc4 protein for SYNV cell-to-cell movement. (A) Schematic representation of rSYNV-GFP-Δsc4:RFP, rSYNV-GFP-ΔM:RFP and rSYNV-GFP-ΔG:RFP recombinant antigenomes in which the RFP gene was substituted for the sc4, M or G gene, respectively. (B) Cell-to-cell movement of rSYNV-GFP and the RFP substitution mutants. *N*. *benthamiana* leaves were agroinfiltrated with plasmids designed to express the indicated agRNA derivatives along with supporting N, P, L and VSR plasmids. Infiltrated leaves were photographed with a fluorescence microscope at 6 dpi and 14 dpi. Scale bar, 200 μm.
(TIF)
