# Supplementary material for: Rescue of a Plant Negative-Strand RNA Virus from Cloned cDNA: Insights into Enveloped Plant Virus Movement and Morphogenesis
Source: PLoS Pathog. 2015 Oct 20;11(10):e1005223. doi: 10.1371/journal.ppat.1005223 (PMC4616665; doi:10.1371/journal.ppat.1005223)
Supplement: S1 Protocols — (DOCX) [file ppat.1005223.s001.docx]

**S1 Protocols. Descriptions of plasmid construction details.**

To generate pGD-NPL for tandem expression of the N, P and L proteins in a pGD binary plasmid [1], the N, P and L expression cassettes were amplified by PCR using the pGD-N, pGD-P and pGD-L as templates, respectively. Each of the three amplified gene cassettes contains the 35S promoter, the gene coding region and the Nos terminator sequence. A separate PCR product was produced by amplification of the pGD backbone sequence to remove the 35S promoter and Nos terminator regions. Each primer designed for these amplifications contains a 15-nt overhang at its 5' end that is homologous to the 5' end of another PCR product, so that the four PCR products could be ligated head-to-tail and circularized in the presence of an In-Fusion Cloning mixture (Clontech, Japan). The primer sequences and detailed annealing information are listed in S1 Table. The assembled plasmid contains the three expression cassettes in the order of N-P-L.

To construct the pSYNV-eGFP plasmid for generation of rSYNV with a GFP expression cassette inserted between the N and P ORF, we used a unique *Nco*I site (CCATGG) in pSYNV that overlaps the start codon (underlined) of the P ORF. The complete coding region of GFP, followed by the N/P gene junction sequence, was amplified from the pSYNV-MR-eGFP-DsRed plasmid [2] by PCR with the primer pairs GFP-NcoI/F and NPJ-NcoI/R (S1 Table). To facilitate sub-cloning, an *Nco*I site was introduced at the ends of the two primers. The resulting PCR product was digested by *Nco*I and ligated into the *Nco*I-digested pSYNV plasmid to generate pSYNV-GFP. Clones with correctly oriented GFP inserts were verified by restriction digestion, and by dideoxynucleotide sequencing.

The pSYNV-GFP-Δsc4, pSYNV-GFP-ΔM, pSYNV-GFP-ΔG and pSYNV-GFP-ΔMG plasmids were derived from pSYNV-GFP by deletion of the entire transcription units (the ORF with flanking gene junction sequences) of sc4, M, G and M plus G, respectively. The gene junctions of SYNV contain a stretch of consensus sequence UAUAAGAAAAA CC AAC (in the antigenome sense), in which the UAUAAGAAAAA likely corresponds to the 3' ends of the upstream mRNAs, and the AAC corresponds to the 5' ends of the downstream mRNAs, while the CC dinucleotide is an non-transcribed intergenic region [3]. Therefore, to delete a given gene, we deleted the entire cDNA region beginning with the upstream CC AAC nucleotides, through the ORF to the downstream UAUAAGAAAAA nucleotides, and joined the two flanking fragments to form a new gene junction sequence. As an example, in the construction of pSYNV-GFP-Δsc4, the unique restriction sites *Nhe*I and *Pml*I were used to facilitate sub-cloning of the upstream P and downstream M ORFs. We amplified the upstream cDNA region encompassing the *Nhe*I site to the 3' end of the P mRNA using the primer pair P-NheI/F and P-sc4 J1/R, and the downstream cDNA region from the 5' end of the M mRNA to the *Pml*I site using primer pair sc4-M J1/F and M-PmlI/R (S1 Table). The two PCR products were joined by over-lapping extension PCR, digested with *Nhe*I and *Pml*I and then the *Nhe*I-*Pml*I fragment in pSYNV-GFP to was substituted to form pSYNV-GFP-Δsc4. Accordingly, the other deletion mutants were generated with a similar approach, using the primers listed in S1 Table and unique restriction sites as indicated in the names of the primers. To construct pSYNV-GFP-ΔM, we used the primers sc4-BstZ/F with sc4-M J1/R and M-G J1/F with G-BstZ/R; to construct pSYNV-GFP-ΔG, the primer M-PmlI/F was used with M-G J1/R and G-L J1/F with L-BstB/R; pSYNV-GFP-ΔMG was constructed with primer sc4-BstZ/F with sc4-M J1/R and G-L J2/F with L-BstB/R (S1 Table).

The pSYNV-GFP-Δsc4:RFP, pSYNV-GFP-ΔM:RFP and pSYNV-GFP-ΔG:RFP plasmids were constructed by using a similar sub-cloning approach as described above, but the sc4, M or G gene coding regions were substituted with the RFP ORF by over-lapping extension PCR. To this end, the upstream and downstream sequences, of a to-be-deleted gene, were amplified with PCR and the RFP ORF substituted into this region. To construct pSYNV-GFP-Δsc4:RFP, we used the P-NheI/F primer with P-sc4 J2/R and sc4-M J2/F with M-PmlI/R; to construct pSYNV-GFP-ΔM:RFP, we used primers the sc4-BstZ/F with sc4-M J2/R and M-G J2/F with G-BstZ/R; to construct pSYNV-GFP-ΔG:RFP, and the primer M-PmlI/F with M-G J2/R and G-L J3/F with L-BstB/R. The primers used for PCR are shown in S1 Table.

To construct pSYNV-MR-sc4-RFP expressing sc4 in the N ORF position and RFP in the P ORF position, we substituted the eGFP sequence in the previously generated pSYNV-MR-eGFP-DsRed [2] with sc4 ORF, by In Fusion cloning using primer pairs MR/F with MR/R, and MR-sc4/F with MR-sc4/R (S1 Table).

**References**

1. Goodin MM, Dietzgen RG, Schichnes D, Ruzin S, Jackson AO. pGD vectors: versatile tools for the expression of green and red fluorescent protein fusions in agroinfiltrated plant leaves. Plant J. 2011; 31: 375-383.

2. Ganesan U, Bragg JN, Deng M, Marr S, Lee MY, Qian S, et al. Construction of a Sonchus Yellow Net Virus minireplicon: a step toward reverse genetic analysis of plant negative-strand RNA viruses. J. Virol. 2013; 87: 10598-10611.

3. Jackson AO, Dietzgen RG, Goodin MM, Bragg JN, Deng M. Biology of plant rhabdoviruses. Annu Rev Phytopathol. 2005; 43: 623-660.
