# Supplementary material for: Rescue of a Plant Negative-Strand RNA Virus from Cloned cDNA: Insights into Enveloped Plant Virus Movement and Morphogenesis
Source: PLoS Pathog. 2015 Oct 20;11(10):e1005223. doi: 10.1371/journal.ppat.1005223 (PMC4616665; doi:10.1371/journal.ppat.1005223)
Supplement: S1 Table — (DOCX) [file ppat.1005223.s002.docx]

**S1 Table. List of primers used in the study.**

| Primer^a^ | Sequence (5' to 3')^b^ | Notes |
| --- | --- | --- |
| 35S-N-Nos/F | ccttatctgggaactACTCACACAT | To amplify the N gene expression cassette; sequence in lower case letters annealed to pGD/R |
| 35S-N-Nos/R | ggagaagcgaactgcATTCCCGATCTAGTAACATAGATGACAC | To amplify the N gene expression cassette; sequence in lower case letters annealed to 35S-P-Nos/F |
| 35S-P-Nos/F | gcagttcgcttctccCCTTATCTGGGAACTACTCACACAT | To amplify the P gene expression cassette; sequence in lower case letters annealed to 35S-N-Nos/R |
| 35S-P-Nos/R | gactctgcctacctcATTCCCGATCTAGTAACATAGATGACAC | To amplify the P gene expression cassette; sequence in lower case letters annealed to 35S-L-Nos/F |
| 35S-L-Nos/F | gaggtaggcagagtcCCTTATCTGGGAACTACTCACACAT | To amplify the L gene expression cassette; sequence in lower case letters annealed to 35S-P-Nos/R |
| 35S-L-Nos/R | attcccgatctagtaACATAGATGACAC | To amplify the L gene expression cassette; sequence in lower case letters annealed to pGD/F |
| pGD/F | tactagatcgggaatTAAACTATCAGTG | To amplify the pGD backbone; sequence in lower case letters annealed to 35S-L-Nos/R |
| pGD/R | agttcccagataaggGAATTAGGGTTC | To amplify the pGD backbone; sequence in lower case letters annealed to 35S-N-Nos/F |
| SYNV/11008/F | TCCTAATAAGTTTCTACC | For identification of genetic tag containing a *Bsm*BI site |
| SYNV/12503/R | CGGAGAGTTGTGAATGTT | For identification of genetic tag containing a *Bsm*BI site |
| GFP-NcoI/F | CATGCCATGGTGAGCAAGGGCGAG | For construction of pSYNV-GFP; *Nco*I site underlined |
| NPJ-NcoI/R | CATGCCATGGCCTAGACAAATAATAC | For construction of pSYNV-GFP*; Nco*I site underlined |
| P-NheI/F | AAGAGAAGGGGCTAGCATGTC | For construction of pSYNV-GFP-Δsc4; *Nhe*I site underlined |
| P-sc4 J1/R | aagttggtttttcttatatAAGTGCACGGAATAATAC | For construction of pSYNV-GFP-Δsc4; sequence in lower case letters annealed to sc4-M J1/F |
| sc4-M J1/F | atataagaaaaaccaacttAAAATAATAATAATATCAAA | For construction of pSYNV-GFP-Δsc4; sequence in lower case letters annealed to P-sc4 J1/R |
| M-PmlI/R | CATCACGACTGACACGTGACCT | For construction of pSYNV-GFP-Δsc4; *Pml*I site underlined |
| sc4-BstZ/F | GCAAGTACTTTGGTATACAAGAAAGG | For construction of pSYNV-GFP-ΔM; *Bst*Z171 site underlined |
| sc4-M J1/R | aagttggtttttcttatatCACACCACAACCATCAC | For construction of pSYNV-GFP-ΔM; sequence in lower case letters annealed to M-G J1/F |
| M-G J1/F | atataagaaaaaccaacttTGATAGTTTATTATTAAG | For construction of pSYNV-GFP-ΔM; sequence in lower case letters annealed to sc4-M J1/R |
| G-BstZ/R | TCTTGAATACTGGTATACTTATTCC | For construction of pSYNV-GFP-ΔM; *Bst*Z171 site underlined |
| M-PmlI/F | AACGACGTAGGTCACGTGTCAGT | For construction of pSYNV-GFP-ΔG; *Pml*I site underlined |
| M-G J1/R | atgttggtttttcttATATAAGTGCACGGAATAATACAGGT | For construction of pSYNV-GFP-ΔG; sequence in lower case letters annealed to G-L J1/F |
| G-L J1/F | aagaaaaaccaacatACATCATCATTTAGT | For construction of pSYNV-GFP-ΔG; sequence in lower case letters annealed to M-G J1/R |
| L-BstB/R | GATGTGACACCAACGTTCGAAT | For construction of pSYNV-GFP-ΔG; *Bst*BI site underlined |
| G-L J2/F | atataagaaaaaccaacttACATCATCATTTAGT | For construction of pSYNV-GFP-ΔMG; sequence in lower case letters annealed to P-sc4 J1/R |
| P-sc4 J2/R | ctcggaggaggccatGATACCTGCATACAGAATATATATAAAAT | For construction of pSYNV-GFP-Δsc4:RFP; sequence in lower case letters annealed to RFP/F |
| sc4-M J2/F | cacctgttcctgtaaCGGCTGGACCTCCGTATTAAG | For construction of pSYNV-GFP-Δsc4:RFP; sequence in lower case letters annealed to RFP/R |
| sc4-M J2/R | ctcggaggaggccatTCTGAAATACAATAGAGATAACCTTG | For construction of pSYNV-GFP-ΔM:RFP; sequence in lower case letters annealed to RFP/F |
| M-G J2/F | cacctgttcctgtaaACCAACCCACCAAAAGCAG | For construction of pSYNV-GFP-ΔM:RFP; sequence in lower case letters annealed to RFP/R |
| M-G J2/R | ctcggaggaggccatTACGAAAAGTTCTTAATAATAAACTATCAAAG | For construction of pSYNV-GFP-ΔG:RFP; sequence in lower case letters annealed to RFP/F |
| G-L J3/F | cacctgttcctgtaaATCCACCCCATAAACACGACC | For construction of pSYNV-GFP-ΔG:RFP; sequence in lower case letters annealed to RFP/R |
| MR/F | ACTACAGCCACAACTCTACCTCC | For construction of pSYNV-MR-sc4-RFP |
| MR/R | GCTAGCGATTACCTGCAATTAAAATAC | For construction of pSYNV-MR-sc4-RFP |
| MR-sc4/F | caggtaatcgctagcATGGAAGGATTATCATCCAAAGC | For construction of pSYNV-MR-sc4-RFP; sequence in lower case letters annealed to MR/R |
| MR-sc4/R | agttgtggctgtagtTCAATAGCTAATGTCGCTCAAC | For construction of pSYNV-MR-sc4-RFP; sequence in lower case letters annealed to MR/F |

^a^ /F: forward primer; /R: reverse primer.

^b^ The sequence shown in lower letters is homologous to other primer as indicated in Notes column, to facilitate In-Fusion cloning; The overlapped restriction site is underlined.
