# Supplementary figures and images for: Rescue of a Plant Negative-Strand RNA Virus from Cloned cDNA: Insights into Enveloped Plant Virus Movement and Morphogenesis
Source: PLoS Pathog. 2015 Oct 20;11(10):e1005223. doi: 10.1371/journal.ppat.1005223 (PMC4616665; doi:10.1371/journal.ppat.1005223)

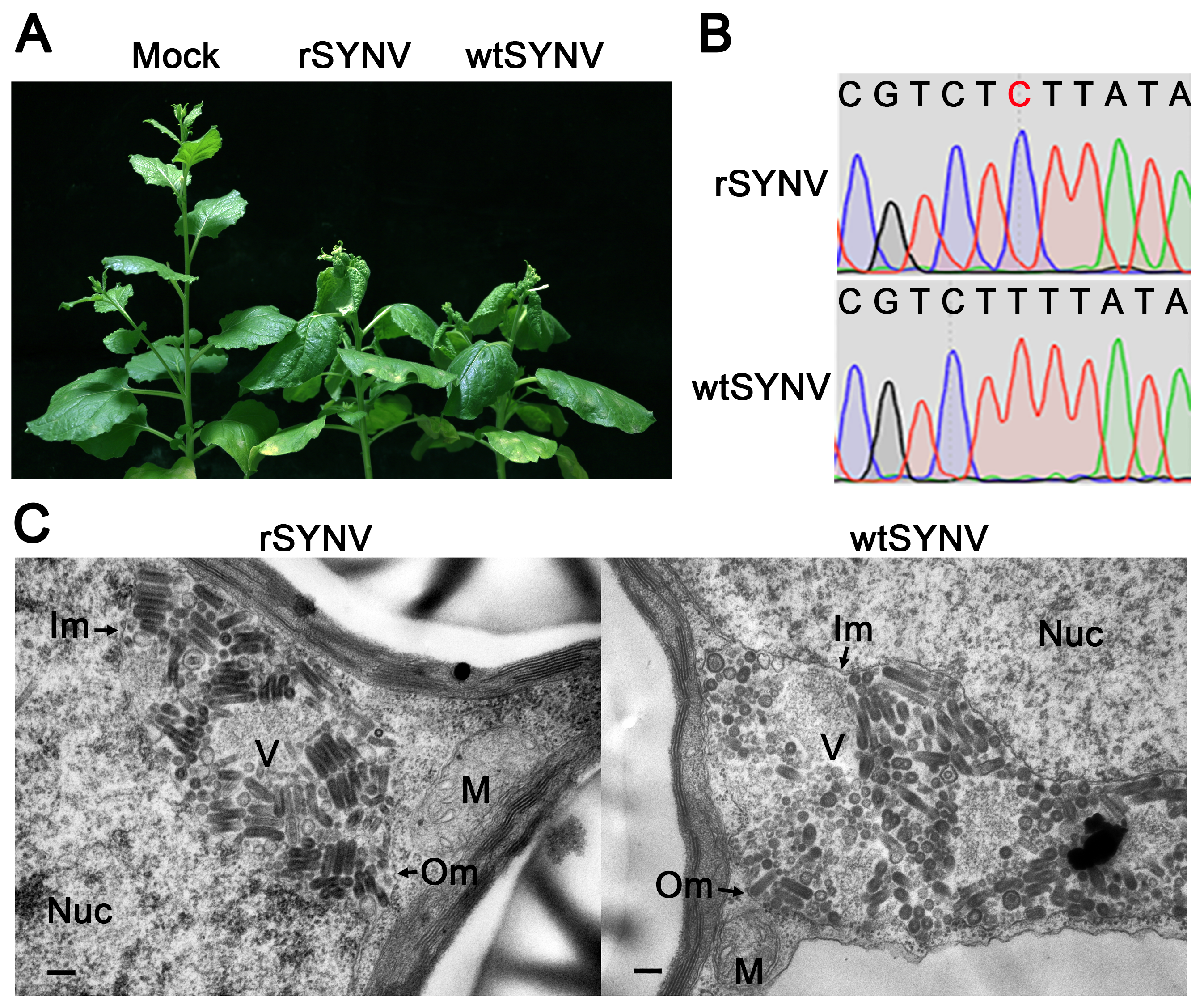

Supplement: S1 Fig — (A) Similar symptom phenotypes were observed at 25 dpi in N. benthamiana plants mechanically inoculated with wtSYNV and rSYNV sap preparations. The rSYNV sap was derived from symptomatic leaves of plants that had been infected by agroinfiltration with SYNV infectious clone. Mock: buffer inoculated plants. (B) Sequence comparison of rSYNV and wtSYNV cDNA regions encompassing the mutation site. Note: The rSYNV and wtSYNV sequences shown above the graphs are presented in the antigenomic sense, and the cytosine mutation (No.13,592) in rSYNV is shown in red. (C) Thin sections of infected plant cells are presented to show the similar cytopathology of cells infected with wtSYNV and rSYNV. In both sections, mature virions (V) were found within perinuclear spaces surrounded by the inner nuclear membrane (Im) and the outer nuclear membrane (Om). Nuc: nucleus; M: mitochondria. Scale bar, 0.2 μm. (TIF) [file ppat.1005223.s003.tif]

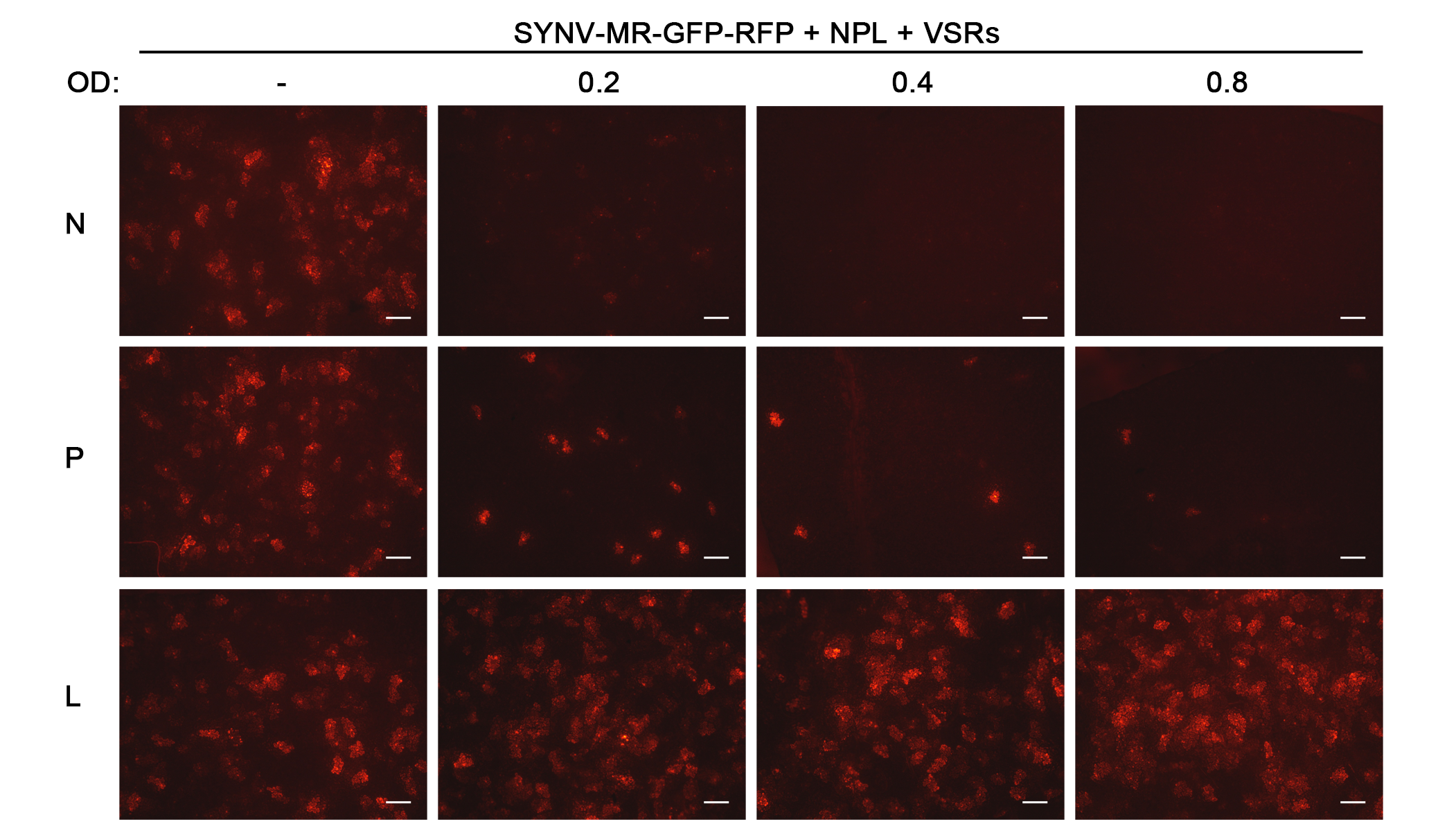

Supplement: S2 Fig — Equal volumes of Agrobacterium cultures at 0.8 OD harboring the SYNV MR-GFP-RFP, pGD-NPL and the three VSRs plasmids were mixed and infiltrated into N. benthamiana leaves. Additional volumes of bacterial cultures containing the pGD-N (upper panels), pGD-P (middle panels) or pGD-L plasmids (bottom panels) at 0.2, 0.4 or 0.8 OD as indicated on the top of panels, were also included in the mixture to test their effects on reporter expression. Infiltrated plants were photographed at 9 dpi with a fluorescence microscope under RFP channel. Scale bar, 200 μm. (TIF) [file ppat.1005223.s004.tif]

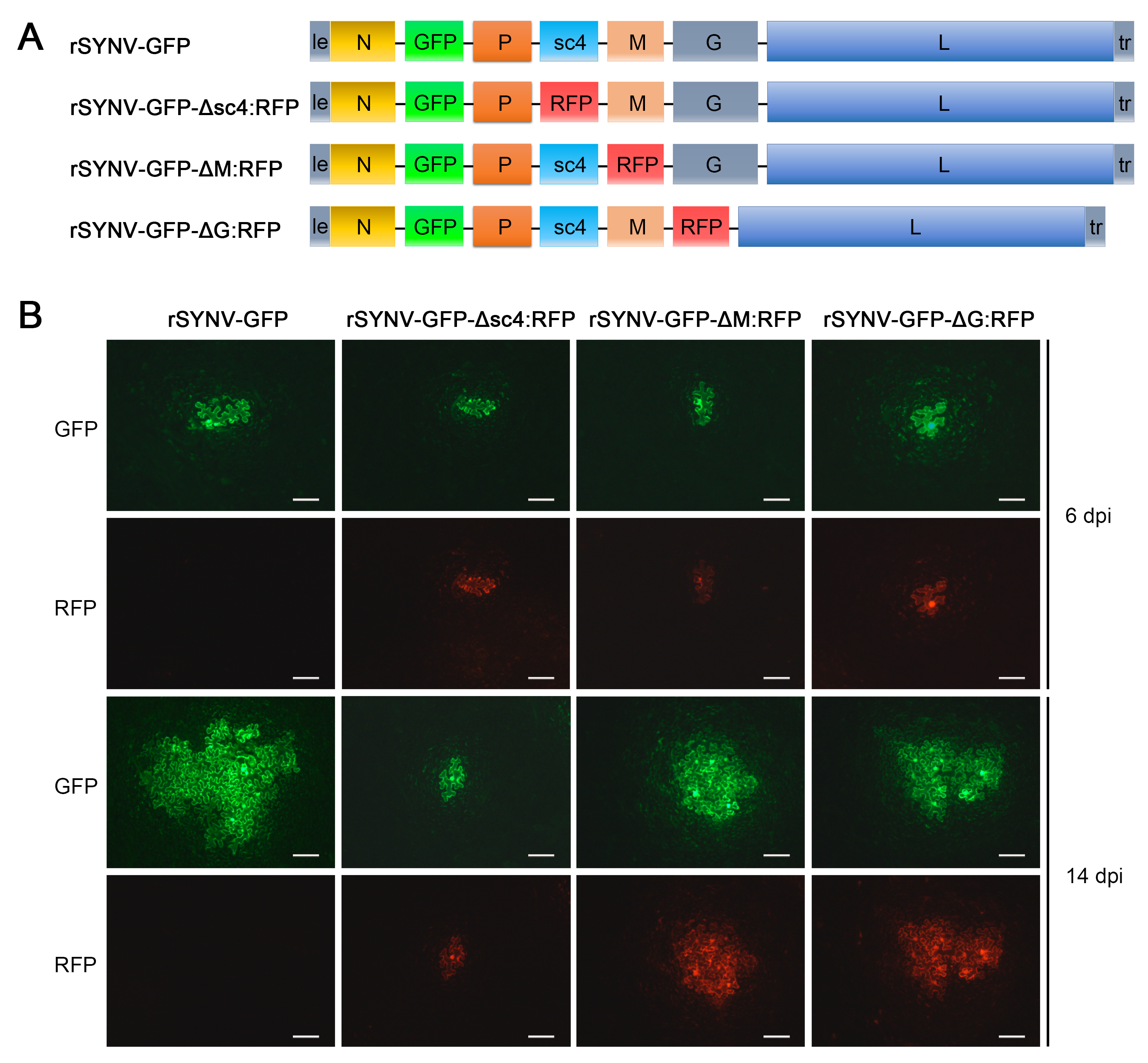

Supplement: S3 Fig — (A) Schematic representation of rSYNV-GFP-Δsc4:RFP, rSYNV-GFP-ΔM:RFP and rSYNV-GFP-ΔG:RFP recombinant antigenomes in which the RFP gene was substituted for the sc4, M or G gene, respectively. (B) Cell-to-cell movement of rSYNV-GFP and the RFP substitution mutants. N. benthamiana leaves were agroinfiltrated with plasmids designed to express the indicated agRNA derivatives along with supporting N, P, L and VSR plasmids. Infiltrated leaves were photographed with a fluorescence microscope at 6 dpi and 14 dpi. Scale bar, 200 μm. (TIF) [file ppat.1005223.s005.tif]
